# Drug Allergy Risk Stratification in Children With Immediate or Delayed Urticaria During Antibiotic Treatment

**DOI:** 10.1111/cea.70181

**Published:** 2025-11-20

**Authors:** Francesca Mori, Leonardo Tomei, Chiara Marzi, Giulia Liccioli, Simona Barni, Mattia Giovannini, Lucrezia Sarti, Benedetta Pessina, Lene Heise Garvey

**Affiliations:** ^1^ Allergy Unit Meyer Children's Hospital IRCCS Florence Italy; ^2^ Department of Statistics, Computer Science and Applications “G. Parenti,” University of Florence Florence Italy; ^3^ Department of Health Sciences University of Florence Florence Italy; ^4^ Allergy Clinic, Copenhagen University Hospital Gentofte Copenhagen Denmark; ^5^ Department of Clinical Medicine University of Copenhagen Copenhagen Denmark

**Summary**

- Assessing the risk for drug hypersensitivity in children who develop urticaria during antibiotic therapy is complex onset.
- The clinical presentation of the reaction should be considered in conjunction with the latency between drug administration and symptom

To the Editor,

In children, the incidence of urticaria is approximately 1% per year, mainly as an acute episode, and among cutaneous adverse reactions to drugs, urticaria is the second most common manifestation after maculopapular exanthemas [1].

Distinguishing between viral infections and drug eruptions is difficult, as the conditions often coexist, and assessing the risk for drug hypersensitivity (DH) in children who develop urticaria during an antibiotic course therapy is complex [2]. However, identifying patients with IgE‐mediated drug‐induced acute urticaria is important because these patients are at risk for severe reactions during a drug provocation test (DPT). Currently, optimizing the drug allergy workup in patients with urticarial reactions represents one of the main controversies between adult and paediatric allergists. In fact, according to proposed risk stratification protocols for adults, acute urticaria after drug intake defines high‐risk patients [3], whereas in the paediatric EAACI Task Force Report, acute urticaria is considered a low‐risk sign [4].

This study aimed to analyze whether the day and the latency between drug intake and onset of urticaria over a course of antibiotic therapy could help to detect patients at higher risk of having a confirmed DH.

In this retrospective study, we included children investigated by the Allergy Unit of Meyer Children's Hospital IRCCS from January 2012 to August 2023 because of isolated urticaria occurring during an antibiotic course therapy. Patients were classified considering the time latency between drug intake and the onset of urticaria and the day of reaction over a course treatment. The diagnosis of drug allergy was correlated with clinical information and time latency, using different multiple logistic regression models. In the subset of patients with a positive DPT, we created contingency matrices to compare information regarding reaction times from the anamnesis and DPT results.

A total of 474 patients were included in the study. Regarding the timing of the index reaction: in 230 patients (48.5%), urticaria appeared during the first day, in 214 (45.2%) over treatment, and in 30 (6.3%) after the last day of antibiotic therapy. Furthermore, in 206 subjects (43.5%), urticaria appeared within an hour, in 170 (35.9%) between 1 and 6 h, and in 98 (20.9%) after 6 h from drug intake. At last, 135 children developed urticaria within the first hour from the first dose of antibiotic and among those 22 (16.3%) were diagnosed with DH.

Overall, DH was confirmed in 68 cases (14.3%): 42 (61.8%) immediate and 26 (38.24%) non‐immediate DH cases. Only one patient (0.2%) had anaphylaxis with amoxicillin/clavulanate DPT (Table 1). No statistically significant difference was detected between the group of patients with confirmed and excluded DH after allergological investigations. However, some trends were noted: patients with a personal history of atopy, with reactions within 1 h from the first dose, with reactions on the first day of the antibiotic course and within 6 h of drug intake had a higher risk for confirmed DH. Additional information about study methods and results can be found at https://doi.org/10.5281/zenodo.17378073.

**TABLE 1:** Demographic and clinical characteristics of the population and results of allergological work‐up.

Age (years)	
Mean age at reaction (SD)	5.1 (3.9)
Gender	
Males; *n* (%)	223 (47.05%)
Females; *n* (%)	251 (52.95%)
Personal history of atopy	110 (23.21%)
Allergic rhinitis; *n* (%)	74 (15.3%)
Asthma; *n* (%)	30 (6.33%)
Atopic dermatitis; *n* (%)	9 (1.90%)
Chronic urticaria; *n* (%)	8 (1.69%)
Food allergy; *n* (%)	4 (0.84%)
Day of therapy course (of onset of reaction)	
1st day; *n* (%)	230 (48.52%)
During the course of treatment; *n* (%)	214 (45.15%)
After the end of therapy; *n* (%)	30 (6.33%)
Time from the last dose	
< 1 h; *n* (%)	206 (43.46%)
1–6 h; *n* (%)	170 (35.86%)
> 6 h; *n* (%)	98 (20.68%)
Confirmed diagnosis *n*;%	68 (14.34%)
Immediate hypersensitivity	42 (61.76%)
Non‐immediate hypersensitivity	26 (38.24%)
Diagnosis confirmed by allergy tests	25 (5.27%)
Positivity of skin tests at immediate reading	11 (16.18%)
Positivity of specific IgE for the culprit drug	9 (13.24%)
Positivity of both in vivo and in vitro tests	5 (7.35%)
Diagnosis confirmed by positive drug provocation test	(9.07%)
Urticaria	(67.44%)
Macular exanthema	(16.28%)
Maculopapular exanthema	(9.98%)
Angioedema	1 (2.33%)
Anaphylaxis*	1 (2.33%)

* Erythematous rash, bronchospasm, and faintness requiring intramuscular adrenaline; in this child, both the specific IgE and skin test results were negative.

To our knowledge, this is the first study that analyzes the risk for confirmatory DH diagnosis in 474 children with antibiotic‐induced urticaria in relation to the timing of its appearance.

A recent paediatric study [5] focusing on benign rashes while on beta‐lactam treatment showed that those with proven DH were mostly children with urticaria and/or angioedema who reacted within 1 h from drug intake. The multiple logistic regression analysis did not identify statistically significant risk factors for positive allergy work‐up; however, some interesting trends suggested that those with a history of immediate reaction are at a higher risk for positive drug allergy work‐up. Additionally, Sabato et al. [6], proved that the 1–1–1 urticaria criterion displayed a sensitivity and specificity of 85%, with negative and positive predictive values of 80% and 90%, respectively in a mostly adult population, because the subgroup analysis for children was not sufficiently powered.

The present study has some limitations: the retrospective design may affect the description of the index reaction because of recall bias [7]; statistical significance in the results was not reached limiting the possibility of providing firm recommendations; the duration of urticaria was not analysed as in the study from Sabato et al.

Recently, the concept of risk stratification has become an important tool for adapting diagnostic strategies to perceived risk and optimizing investigations in terms of efficiency, resources and safety.

Although no firm recommendations can be obtained from this study, the trends suggested that patients with urticaria within 6 h on the first day of treatment or within 1 h during a drug course therapy were more likely to have a positive allergy work‐up. In addition, skin tests were frequently positive in children with a history of immediate reactions (38%), suggesting to reserve in vivo tests for that subgroup and to directly provoke all the others.

In a recently published literature review of institution‐specific criteria for low‐risk penicillin allergy, which included both only adult (32 studies) and only paediatric (18 studies) populations [8], the presence of urticaria was included as a feature of low‐risk penicillin allergy in two out of six paediatric studies (33%) and not in any adult studies. Moreover, latency since the index reaction was included in 75% (6/8) of the adult studies, and in no paediatric studies.

To improve risk stratification in children, we propose combining the clinical reaction with the latency between drug administration and symptom onset, and not categorizing urticaria by itself as a risk factor for true DH. Thus far, larger clinical multicenter prospective studies are required to better define the risk factors and further improve drug allergy workup in children.

## Author Contributions

F.M., L.T. and L.H.G. conceptualised and designed the work. L.T. acquired the data. C.M. analysed the data. F.M. and L.T. drafted the initial manuscript. C.M., G.L, S.B., M.G., L.S., B.P., L.H.G. reviewed the manuscript. All authors have read and agreed to the published version of the manuscript.

## Funding

This work was supported, in part, by funds from the ‘Current Research Annual Funding' of the Italian Ministry of Health.

## Ethics Statement

The study was conducted in accordance with the Declaration of Helsinki and approved by the Institutional Review Board statement of Meyer Children's Hospital IRCCS.

## Consent

Written informed consent was obtained from the children's parents for all procedures performed and for publication.

## Conflicts of Interest

The authors declare no conflicts of interest. M.G. reports personal fees from Sanofi, Thermo Fisher Scientific.

## Data Availability

The data that support the findings of this study are available from the corresponding author upon reasonable request.
